# Molecular insights into the dynamic relationship between respiration rate and sulfur isotope effect

**DOI:** 10.1128/aem.01064-25

**Published:** 2025-10-16

**Authors:** Dong Kyun Woo, Bokyung Kim, Yuichiro Ueno, Shawn Erin McGlynn, Min Sub Sim

**Affiliations:** 1 School of Earth and Environmental Sciences, Seoul National University 542392, Gwanak-gu, Seoul, South Korea; 2 Department of Earth and Planetary Sciences, Institute of Science Tokyo 595285 , Meguro-ku, Tokyo, Japan; 3 Earth-Life Science Institute, Institute of Science Tokyo 13290, Meguro-ku, Tokyo, Japan; 4 Blue Marble Space Institute of Science 824783, Seattle, Washington, USA; 5 Biofunctional Catalyst Research Team, RIKEN Center for Sustainable Resource Science, Wako, Japan; Georgia Institute of Technology, Atlanta, Georgia, USA

**Keywords:** microbial sulfate reduction, multiple sulfur isotopes, nitrogen fixation, gene expression, ATP/AMP ratio

## Abstract

Microbial sulfate reduction and its resulting sulfur isotope effects are crucial for understanding the past and present sulfur cycle. Microbial S-isotope effects have often been explained by their inverse correlation with the cell-specific sulfur reduction rate (csSRR), but exceptions exist. A notable example is when N_2_-fixing sulfate reducers fractionate sulfur isotopes more than those cultivated under fixed nitrogen, despite having a faster csSRR. To further understand the biochemical basis of the csSRR and S-isotope fractionation relationship, we monitored gene expression, ATP/AMP ratio, and triple S-isotope fractionation of a sulfate-reducing bacterium, DMSS-1 (*Desulfovibrio* sp.), under ammonium-repleted or depleted conditions with various electron donors. N_2_ fixation with malate occurred with an elevated metabolic sulfur flux as indicated by enhanced sulfate reduction gene expression, but also with an increased reversibility of sulfate activation and reduction, evidenced by a reduced ATP/AMP ratio. The two simultaneous molecular alterations potentially explain the higher fractionation values observed under N_2_ fixation. Meanwhile, N_2_ fixation with fructose, a more refractory but energy-rich electron donor, did not alter gene expression or ATP/AMP ratio, but led to increased csSRR and decreased isotope fractionation. Integrating our findings into the ^32^S, ^33^S, and ^34^S sulfur isotope model suggests that N_2_ fixation reduces leakage of sulfate across the cell membrane during fructose catabolism. Gene expression analysis of carbon catabolism indicates that elevated intracellular NADH may underlie this contrasting response to N_2_ fixation. While the csSRR and S-isotope fractionation relationship is demonstrably dynamic in this study, it reinforces the robustness of the typical inverse correlation in most natural environments, where csSRR high enough to overturn this relationship are rarely observed.

**IMPORTANCE:**

Sulfate-reducing microorganisms produce sulfide depleted in heavy sulfur isotopes during respiration, making the distribution of sulfur isotopes in natural environments an important clue for tracing their activity and physiology. An apparent inverse correlation between cell-specific respiration rate and sulfur isotope fractionation has been widely accepted as a primary control on naturally occurring sulfur isotope signatures. However, exceptions to this trend have been reported, warranting a better mechanistic understanding. Here, using the model sulfate-reducing bacterium DMSS-1, we manipulated carbon and nitrogen sources and monitored sulfur isotope fractionation, respiratory gene expression, and cellular energy status to provide a molecular and biochemical basis for the dynamic relationship between respiration rates and isotope effects. While this relationship is variable, our results suggest that reversing the inverse trend requires exceptionally fast respiration rates rarely achieved in natural environments. This highlights the robustness of the conventional inverse relationship in nature, despite intracellular complexity.

## INTRODUCTION

Microbial sulfate reduction (MSR) plays a crucial role in both the past and present biogeochemical cycles of sulfur and carbon. Its activities of remineralizing organic carbon and reducing sulfur compounds in marine sediments are some of the major fluxes that shaped the sulfur and carbon cycle (1–3). Through this biochemical process, sulfur isotopes can fractionate, where the resulting sulfide becomes depleted of heavier isotopes compared to the remaining sulfate. These isotopic signatures can be preserved in the sedimentary records in various forms, such as carbonate-associated sulfate or pyrite, allowing the reconstruction of the sulfur cycle and metabolic activities of MSR long after their cessation. MSR and its associated sulfur isotope effects have been studied for several decades, and many of these studies were based on laboratory pure cultures of sulfate-reducing microbes (SRM) (4–7). Numerous fundamental insights have been revealed through these studies, inspiring research on microbial isotope fractionation of other oxyanions, including nitrate (8), selenate (9), and uranium (VI) (10). Furthermore, mathematical models have also been proposed to explain and predict the magnitude of microbial sulfur isotope fractionation (11–13). More recently, Wing and Halevy (14) introduced a series of biological parameters into their model, but many of these have yet to be experimentally validated across a range of growth conditions.

One of the major factors that has been reported to control the magnitude of sulfur isotope effects is the cell-specific sulfur reduction rate (csSRR) (5, 15, 16), which represents the amount of sulfur reduced by a single cell in a given time. Numerous studies have shown that the two are inversely correlated; hence, csSRR has been widely used as a parameter to explain the observed sulfur isotope fractionation in environmental samples (3, 17). However, several exceptions to this relationship have been reported. One such example is diazotrophic growth (18). During N_2_ fixation, cells experience an increased demand for ATP because N_2_ reduction by the nitrogenase enzyme couples electron transfer with ATP hydrolysis (19). While this high energy demand necessitates faster respiration, diazotrophic cells fractionated sulfur isotopes to a greater extent, demonstrating that csSRR may not always be a reliable indicator of the magnitude of microbial sulfur isotope fractionation (18).

N_2_-fixing sulfate-reducing microorganisms may have a significant impact on the natural abundance of sulfur isotopes in the marine sedimentary environment. In theory, fixed nitrogen sources such as NH_4_^+^ are the energetically preferred option for most organisms, and the presence of these compounds typically represses biological nitrogen fixation due to its high metabolic cost (20, 21). However, an increasing number of SRM have been found to possess the genetic capacity for N₂ fixation, and *nif* genes affiliated with SRM have been widely detected in anoxic marine sediments, where NH₄^+^ often accumulates as a byproduct of organic matter degradation (22). Beyond genetic potential, inhibition experiments using molybdate, which selectively blocks sulfate reduction, have concurrently suppressed N_2_ fixation in marine sediments (23, 24). These findings suggest that, contrary to conventional expectations, SRM may retain N_2_ fixation activity even in ammonium-rich benthic environments (22). As N_2_ fixation by SRM appears more widespread than previously thought, its influence on microbial sulfur isotope fractionation warrants closer investigation.

Recent investigations into SRM and their sulfur isotope effects have advanced from molecular, biochemical, and metabolic perspectives, through approaches such as knocking out or perturbing key genes (25–27), measuring the concentrations and isotopic compositions of intracellular sulfur metabolites (28), purifying and characterizing individual sulfur-reducing enzymes (29, 30), and profiling the entire proteome of sulfate-reducing bacterium (7). Despite these accomplishments, the deviation of sulfur isotope fractionation from the expected csSRR trend during diazotrophic growth remains enigmatic. To identify the biochemical basis underlying this deviation, we investigate sulfur isotope fractionation by SRM under varying electron donor types and fixed nitrogen concentrations, primarily focusing on intracellular energy states and the expression levels of key metabolic enzymes.

## MATERIALS AND METHODS

### Batch culture experiment

Sulfate-reducing strain DMSS-1 was incubated in a chemically defined, carbonate-buffered medium containing (per liter) NaHCO_3_, 9 g; Na_2_SO_4_, 3 g; KH_2_PO_4_, 0.2 g; NaCl 21 g; MgCl_2_·6H_2_O, 3.1 g; KCl, 0.5 g; CaCl_2_·2H_2_O, 0.15 g; resazurin, 1 mg; 1 mL of trace element solution SL-10 (31); 10 mL of vitamin solution described as a part of DSMZ medium 141 (catalog of strains 1993; DSMZ, Braunschweig, Germany); and 1 mL of tungsten-selenium stock solution (4 mg of Na_2_WO_4_·2H_2_O and 3 mg of Na_2_SeO_3_·5H_2_O per 1 L of 0.01 M NaOH). Ascorbic acid (1 g per liter) and titanium (III) chelated by nitrilotriacetate (60 µM) were used as reducing agents. Cultures contained either lactate (20 mM), malate (20 mM), or fructose (10 mM) as electron donors and carbon sources. DMSS-1 incompletely oxidizes these substrates to acetate, as shown in the following reactions with calculated free energy released assuming standard conditions (pH = 7, *T* = 298.15 K, aqueous concentration of reactants and products aside from H^+^ = 1 M):

2Lactate^-^ + SO_4_^2-^ → 2acetate^-^ + 2HCO_3_^-^ + HS^-^ + H^+^$\Delta G^{{}^{\circ }}\prime$ (kJ/reaction) = −160.1

2Malate^2-^ + SO_4_^2-^ + 2H_2_O → 2acetate^-^ +4HCO_3_^-^ + HS^-^ + H^+^$\Delta G^{{}^{\circ }}\prime$ (kJ/reaction) = −204.9

Fructose + SO_4_^2-^ → 2acetate^-^ +2HCO_3_^-^ + HS^-^ +3H^+^$\Delta G^{{}^{\circ }}\prime$ (kJ/reaction) = −360.1

Electron donor concentrations were selected based on these stoichiometric requirements to ensure that sulfate remained in excess and did not become growth-limiting. For ammonium-replete growth cultures (control), 0.3 g per liter of NH_4_Cl was added before sterilization. Completed media were titrated to pH 7.5 and sterilized anaerobically under 80% N_2_–20% CO_2_ gas. Cells were washed three times by anaerobic centrifugation and resuspended in fresh medium without NH_4_Cl to minimize the carryover of sulfide and NH_4_^+^. All the batch culture experiments were triplicated and conducted at 25°C.

For the experiment with lactate, 30 mL of completed sterile medium was poured into 40 mL culture bottles. At each sampling point, 2 and 10 mL of the inoculated medium were used for measuring growth (optical density, cell count, and sulfide and sulfate concentration) and extracting RNA, respectively. The remaining culture was mixed with 3 mL of anaerobic 1 M Zn-acetate to terminate microbial activity and fix remaining sulfide for sulfur isotope measurements. Fixed sulfide samples were stored at 4°C until further processing. Sampling was conducted every 24 hours from the day of inoculation, and each sampling point was triplicated by sacrificing three separate bottles.

For the experiment with malate and fructose, completed sterile medium was poured into 1 L bottles. Each experiment was triplicated (three for NH_4_^+^ growth and three for N_2_ fix growth) and was monitored every 2 days from the same bottles. At each sampling point, 2 and 8 mL of culture samples were used for measuring cell growth (optical density, cell count, and sulfide and sulfate concentration) and extracting RNA, respectively. An additional 10 mL was taken out and mixed with 3 mL of 1 M Zn-acetate in a separate 50 mL conical tube for sulfur isotope analysis. These samples were stored at 4°C until sulfur extraction for isotope analysis.

Sulfide concentrations were measured by fixing a 200 µL culture sample with 1 mL of 0.05 M Zn-acetate solution. A modified version of the methylene blue assay (32) was used to measure these samples. The same fixed samples were centrifuged, and their supernatant was extracted for measuring the remaining SO_4_^2-^ concentration by Dionex Aquion ion chromatography with an AS11 column (Dionex, Sunnyvale, CA, USA). Growth was monitored by measuring the optical density at 660 nm using a Synergy Biotek microplate reader (Biotek, Winooski, VT, USA) and by microscopic counts of cells utilizing SYTOX-Green nucleic acid stain (Invitrogen, Paisley, UK) and an Echo Revolve 4 epifluorescence microscope (Echo, San Diego, CA, USA). All samples were stored at 4°C before further processing.

### Total RNA extraction

Culture samples taken for RNA extraction were transferred into 2 mL centrifuge tubes. The tubes were centrifuged, and approximately 1.8 mL of the supernatant was discarded. The remaining cell pellets were resuspended and combined into a single 2 mL tube. The newly combined culture samples were again centrifuged, and the supernatant was completely taken out and replaced with 1 mL of RNAlater solution (Invitrogen, Paisley, UK) for RNA preservation. The preserved samples were stored at 4°C until further extraction process.

Total RNA extraction was conducted using Purelink RNA Mini Kit with TRIzol solution (Invitrogen, Paisley, UK) as a cell lysis reagent. Before using the extraction kit, samples were centrifuged, and the supernatants were removed completely. TRIzol solution was added to the remaining cell pellet, and further extraction was conducted according to the manual of the kit, including the optional DNase treatment steps. The resulting concentrations of RNA samples were quantified using QuantiFluor RNA System (Promega, Madison, WI, USA), and the purity of these samples, including *A*_260_/*A*_280_, was measured using Nanodrop One^C^ spectrophotometer (Thermo Fisher Scientific, Waltham, MA, USA). Only samples with a sufficient amount (>0.5 ng/µL) and quality (*A*_260_/*A*_280_ > 1.8) of RNA were used for further experiments.

### Relative quantification of gene expression levels using RT-qPCR analysis

Two major sulfur-reducing genes were targeted for RT-qPCR analysis: adenylyl-sulfate reductase (*aprA*) and dissimilatory sulfite reductase (*dsrA*) gene. In addition, to observe the potential difference in the consumption of fructose during nitrogen-depleted experiments, three major genes involved in glycolysis were monitored: pyruvate kinase (*pyk*), phosphofructokinase (*pfk*), and glyceraldehyde 3-phosphate dehydrogenase (*gap*). Reverse transcription was performed with a High-Capacity cDNA Reverse Transcription Kit (Applied Biosystems, Waltham, MA, USA). Seventy-five nanograms of RNA was used per reverse transcription reaction with random hexamers and RNase inhibitor given by the manufacturer. The resulting cDNA was quantified using QuantStudio 3 (Applied Biosystems, Waltham, MA, USA), with a reaction mixture of 10 µL of Power Up SYBR Green qPCR Master Mix (Applied Biosystems, Waltham, MA, USA), 1 µL of primer mix (details shown in Table S1), and 7 µL of PCR-grade distilled water to a final volume of 20 µL. Thermal procedure and primer information for each target gene are explained in Table S1. The resulting amplicons were run with gel electrophoresis to confirm the expected base pair length (results not shown). Raw data from the qPCR analysis were processed using LinRegPCR software in order to calculate the initial cDNA quantity (N0) using the average PCR efficiency of each individual reaction (33, 34). The resulting N0 values, which are expressed in arbitrary fluorescence units, were used to compare the effects of different medium compositions. The N0 values of the 16s rRNA gene were used to normalize the expression level of the target genes in order to correct the data from potential differences in RNA quantity and RT-qPCR efficiency between samples. Each RNA sample was analyzed three times, and the resulting standard deviation was used as the error value.

### Intracellular ATP/AMP ratio

Cell samples from the malate and fructose experiments were disrupted and deproteinized prior to analysis. For preparation, trichloroacetic acid (TCA) was diluted to 10% using ice-cold distilled water, and diethyl ether was presaturated by mixing an equal volume of Tris-EDTA (TE) buffer in a glass bottle under room temperature. Several million cells were pelleted in a microcentrifuge tube, and the supernatant was discarded. The cell pellet was resuspended in 0.2 mL of ice-cold 10% TCA, and the mixture was homogenized. The homogenized sample was incubated on ice for 30 minutes and then centrifuged at 13,000 rpm for 5 minutes. The supernatant was transferred to a new microcentrifuge tube, and an equal volume of TE-saturated ether was added. The mixture was vortexed for 20 seconds. The tube was then spun for 10 seconds, and the ether layer was carefully removed from the top. This process of adding and removing saturated ether was repeated twice. Finally, the tube was left uncapped in a fume hood for 30 minutes to allow any residual ether to evaporate. Deproteinized samples were stored at −80°C until further use.

ATP/AMP ratios were determined using an ATP/ADP/AMP Assay Kit (Biomedical Research Service) following the manufacturer’s instructions. In this assay, the enzyme luciferase oxidizes luciferin in the presence of ATP, resulting in light emission. ATP levels in the samples were measured using a Synergy microplate reader (Biotek, Winooski, VT, USA), and ADP and AMP concentrations were determined in the same manner after enzymatic conversion to ATP. Since we found that cell disruption yields varied considerably, the subsequent discussion focuses solely on the ATP/AMP ratio, rather than on the absolute concentrations of ATP or AMP. The uncertainty of the ATP/AMP ratio was calculated based on the standard deviations of at least five replicate measurements for prepared standards and samples obtained from cultures.

### Sulfur isotope measurements

Sulfide samples were extracted under a stream of N_2_ gas (99.999%) by boiling them with 6 N of HCl for 2 hours using a heating mantle that was set to 120°C. The volatile sulfide was trapped with 100 mL of AgNO_3_ solution, which contained about 0.18 M of AgNO_3_ with 1 L of 10% NH_4_OH. After the extraction of sulfide, the remaining sample solution was further reacted with an acid mixture solution containing HI, H_3_PO_2_, and HCl according to Thode et al. (35) in order to extract the remaining sulfate. Volatile products were passed through a 4°C condenser and a trap containing distilled water and ultimately trapped in an AgNO_3_ solution. The extracted sulfide and sulfate were converted into Ag_2_S, and after a day of incubation at 60°C, Ag_2_S samples were centrifuged, washed with distilled water three times, and dried at 60°C. The collected Ag_2_S samples were then converted into SF_6_ gas by using CoF_3_ and a Curie-Point pyrolyzer (36). SF_6_ samples were purified using an automated vacuum line and transferred to an isotope-ratio mass spectrometer for multiple sulfur isotope measurements (37). All the triplicated sulfate samples from the NH_4_^+^-replete lactate batch culture experiment were analyzed to confirm that the isotope data from the rest of the batch cultures were consistent (Fig. S1). Hence, the isotope data shown in this study are from one of the triplicated culture experiments.

### Data processing

Growth yield (number of cells/mol of SO_4_^2-^ reduced) was calculated using the ratio of increase in the number of cells and in the sulfide produced (Table 1)


$$Growth\;yield=\;\frac{C_{N}-C_{1}}{{\left[H_{2}S\right]}_{N}-{\left[H_{2}S\right]}_{1}}.$$

**TABLE 1:** Growth and isotope data from batch culture experiments^*d.*^

Electron donor	Time (day)	Cell density (cells/mL)^*a*^	Sulfide (mM)^*a*^	Sulfate (mM)^*a*^	*f*	Growth yield (10^6^ cells/µmol of SO_4_^2-^)^*b*^	csSRR (fmol/cell/ day)^*b*^	Sulfide (‰)^*c*^		Sulfate (‰)^*c*^		^34^ε (‰)^*b*^	^33^E (‰)^*b*^	^33^λ^*b*^
								δ^33^S	δ^34^S	δ^33^S	δ^34^S			
Lactate (NH_4_^+^)	0		0.00	21.52	1.00					−2.11	−4.06			
	2	3.12 × 10^7^	6.33	17.68	0.82			−5.01	−9.81	−1.58	−3.08	6.1 ± 0.2	−0.043 ± 0.013	0.5080 ± 0.0021
	3	1.76 × 10^8^	9.58	11.56	0.54	18.82 ± 2.90	31.3 ± 6.9	−4.46	−8.74	−0.60	−1.18	5.7 ± 0.2	−0.034 ± 0.011	0.5091 ± 0.0019
	4	1.35 × 10^8^	8.87	11.39	0.53	15.10 ± 2.34	15.2 ± 3.8	−4.63	−9.04	−0.06	−0.10	6.6 ± 0.2	−0.035 ± 0.011	0.5098 ± 0.0016
Lactate (N_2_ fix)	0		0.02	21.04	1.00					−1.83	−3.58			
	2	9.08 × 10^6^	0.34	19.36	0.92									
	4	2.98 × 10^7^	3.23	17.80	0.85	7.18 ± 1.67	75.6 ± 9.5	−5.13	−10.00	−1.45	−2.86	6.6 ± 0.2	−0.009 ± 0.013	0.5137 ± 0.0020
	5	5.74 × 10^7^	9.70	12.43	0.59	5.24 ± 0.95	148.3 ± 20.2	−5.11	−10.05	0.10	0.20	8.0 ± 0.2	−0.066 ± 0.011	0.5068 ± 0.0014
	6	6.74 × 10^7^	10.33	11.66	0.55	5.38 ± 0.95	73.0 ± 10.0	−5.01	−9.82	0.52	1.02	8.2 ± 0.2	−0.052 ± 0.011	0.5088 ± 0.0013
	8	9.80 × 10^7^	10.62	11.76	0.56	8.70 ± 1.45	28.9 ± 4.1	−4.99	−9.77	0.51	0.99	8.2 ± 0.2	−0.043 ± 0.011	0.5098 ± 0.0013
Malate (NH_4_^+^)	0		0.01	23.13	1.00					−1.48	−2.90			
	7	2.75 × 10^7^	0.34	22.66	0.98									
	9	2.27 × 10^8^	1.95	19.87	0.86	114.89 ± 17.64	6.4 ± 0.9	−10.90	−21.26	−0.96	−1.89	18.1 ± 0.5	−0.089 ± 0.014	0.5101 ± 0.0007
	11	5.97 × 10^8^	6.43	16.95	0.73	92.42 ± 14.06	5.8 ± 0.8							
	13	6.44 × 10^8^	8.65	13.60	0.59	74.04 ± 11.26	3.7 ± 0.5							
	15	6.75 × 10^8^	8.33	13.21	0.57	81.03 ± 12.26	2.3 ± 0.3							
Malate (N_2_ fix)	0		0.00	22.99	1.00					−1.65	−3.23			
	7	1.26 × 10^7^	0.36	22.81	0.99									
	9	4.10 × 10^7^	0.81	21.94	0.95	50.89 ± 7.70	8.3 ± 1.3							
	11	9.24 × 10^7^	2.40	21.26	0.92	39.20 ± 6.92	9.7 ± 1.4	−11.92	−23.20	−1.20	−2.34	20.2 ± 0.5	−0.089 ± 0.014	0.5106 ± 0.0007
	13	1.79 × 10^8^	4.77	19.04	0.83	37.90 ± 6.18	7.7 ± 1.2							
	15	2.23 × 10^8^	6.22	17.60	0.77	35.92 ± 5.77	6.2 ± 0.9							
	17	2.24 × 10^8^	6.61	17.62	0.77	33.89 ± 5.13	5.3 ± 0.8							
Fructose (NH_4_^+^)	0		0.00	21.00	1.00					−1.04	−2.03			
	13	3.11 × 10^7^	0.16	20.26	0.96									
	15	1.33 × 10^8^	0.95	19.86	0.95	127.00 ± 26.20	2.6 ± 0.4							
	17	4.56 × 10^8^	3.11	18.38	0.88	144.62 ± 23.62	3.3 ± 0.5	−20.13	−38.95	0.51	0.95	37.0 ± 0.9	−0.108 ± 0.015	0.5121 ± 0.0004
	19	1.13 × 10^9^	8.68	14.73	0.70	129.72 ± 20.19	3.5 ± 0.5							
	21	1.03 × 10^9^	8.36	14.34	0.68	122.44 ± 19.10	1.8 ± 0.3	−15.74	−30.57	3.68	7.11	31.2 ± 0.7	−0.105 ± 0.012	0.5117 ± 0.0004
Fructose (N_2_ fix)	0		0.00	21.87	1.00					−1.02	−1.99			
	21	4.66 × 10^7^	0.77	20.36	0.93									
	23	8.54 × 10^7^	1.33	20.08	0.92	52.31 ± 9.78	4.3 ± 0.8							
	25	1.58 × 10^8^	2.21	19.29	0.88	68.54 ± 11.49	3.5 ± 0.5	−18.32	−35.52	−0.87	−1.70	31.9 ± 0.8	−0.130 ± 0.015	0.5109 ± 0.0004
	27	2.64 × 10^8^	3.81	17.97	0.82	65.45 ± 10.51	3.3 ± 0.5	−16.50	−32.06	0.21	0.37	29.6 ± 0.7	−0.119 ± 0.014	0.5110 ± 0.0004
	29	4.22 × 10^8^	5.97	16.27	0.74	68.58 ± 10.75	2.8 ± 0.4	−13.02	−25.33	1.28	2.45	23.8 ± 0.6	−0.083 ± 0.013	0.5115 ± 0.0004
	31	5.29 × 10^8^	10.18	13.04	0.60	50.30 ± 7.83	3.3 ± 0.5	−10.67	−20.77	2.53	4.90	20.1 ± 0.5	−0.062 ± 0.011	0.5119 ± 0.0004

^*a*^ Analytical uncertainties of cell density, sulfide, and sulfate concentrations are ±15%, ±2%, and ±5%, respectively.

^*b*^ Error propagation is based on the analytical uncertainty of cell densities, sulfide concentrations, and isotope measurements. The results of growth yield, csSRR, and isotope fractionation values are accumulative up to the day of sampling.

^*c*^ Reproducibility of the isotope data was confirmed from the triplicated sulfate isotope data of NH_4_^+^-replete lactate experiment.

^*d*^ Growth parameter results are average values obtained from triplicated batch culture experiments, while isotope results are from one of the three culture experiments.

*C_1_* and *C_N_* are cell densities (number of cells/mL) at initial and *N*th sampling time, respectively. The average cell-specific sulfate reduction rates were calculated as


$$csSRR=\frac{{\left[H_{2}S\right]}_{N}-{\left[H_{2}S\right]}_{1}}{\sum_{n=1}^{N-1}\frac{C_{n}+C_{n+1}}{2}\times \left(t_{n+1}-t_{n}\right)}\;,$$

where *t_n_* is the time of sampling. The equation shown here is based on previous studies (5, 18), where more than two cell density data are used to reflect a more realistic growth.

Sulfur isotopic compositions are reported using the conventional delta notation


$$\delta^{x}S=1,000\times \left(\frac{{}^{x}R_{\text{sample }}}{{}^{x}R_{\text{reference }}}-1\right),$$

where *^x^R* represents isotopic ratios (*^x^S/^32^S*, where *x* = 33 or 34) of an unknown sample or reference material. For this study, a laboratory in-house SF_6_ gas was used as the reference material. It was calibrated against the IAEA S-1 and S-2 international standards and has δ^33^S and δ^34^S values of −3.92‰ ± 0.16‰ and −7.67‰ ± 0.33‰, respectively, on the VCDT scale (37). Isotope fractionation factor (α) in batch culture experiments was calculated using the Rayleigh distillation equation, assuming constant mass balance between sulfate and sulfide during MSR (5).


$${}^{x}\alpha =-\frac{1}{\ln f_{r}}\ln\left(1+\frac{\left(1-f_{r}\right)}{f_{r}}\times \frac{\delta^{x}S_{p}+1,000}{\delta^{x}S_{r}+1,000}\right),$$

where *f_r_* is the fraction of the remaining sulfate, and *δ*^*x*^S*_p_* and *δ*^*x*^S*_r_* are sulfur isotope compositions (where *x* = 33 or 34) of the sulfide produced between the day of inoculation and the day of sampling and the sulfate remaining at the day of sampling, respectively. The resulting isotope enrichment factor is defined as


$${}^{x}\varepsilon =1,000\times \left(1-{}^{x}\alpha \right).$$

The definition of *^x^ε* described here means that positive values represent the depletion of heavy isotopes in the product.

The fractionation of ^33^S by sulfate reduction is represented as ^33^E, which represents the degree of deviation from the predicted isotope value based on the thermodynamic equilibrium at low temperature. ^33^E is defined as:


$${}^{33}E=-1,000\times \left({}^{33}\alpha -{}^{34}\alpha {}^{0.515}\right)\approx {}^{33}\varepsilon -0.515\times {}^{34}\varepsilon .$$

Non-zero ^33^E values express deviation of ^33^α with respect to the predicted mass-dependent fractionation.

Additionally, ^33^λ values can also be used to represent the fractionation of ^33^S, and it is defined as:


$${}^{33}\lambda =\frac{\ln\left({}^{33}\alpha \right)}{\ln\left({}^{34}\alpha \right)}\approx \frac{{}^{33}\varepsilon }{{}^{34}\varepsilon }.$$

While both notations are commonly used to represent ³³S fractionation, we adopt ³³E in the following discussion because it is more easily implemented in δ³³S vs Δ³³S space and maintains relatively stable uncertainty across a wide range of ³⁴ε values.

The errors of *^x^ε*, ^33^E, and ^33^λ values were calculated based on the propagation of error values from isotope analysis (0.07‰ for δ^33^S, 0.13‰ for δ^34^S, and 0.01‰ for δ^33^S-0.515 δ^34^S), sulfate (±5%), and sulfide (±2%) concentration measurements.

## RESULTS

### Growth characteristics of DMSS-1 depending on different culture conditions

DMSS-1 grown on numerous conditions (different types of electron donors and/or levels of NH_4_^+^ concentration) resulted in sulfate to sulfide reduction occurring at various rates. The resulting csSRR values were between 1.8 and 148.3 fmol/cell/day (Table 1). DMSS-1 growing on lactate showed the fastest csSRR with the lowest growth yield (number of cells/mol of SO_4_^2-^ reduced), while the experiment with fructose resulted in the slowest csSRR with the largest growth yield. The experimental results with malate as an electron donor ranged between 2.3 and 9.7 fmol/cell/day for csSRR and 33.89 × 10^6^ to 114.8 × 10^6^ cells/µmol of SO_4_^2-^ reduced for growth yield, falling generally in between those of lactate and fructose. These results are similar to those of a previous study of DMSS-1 (5), indicating the consistency of our procedures. Different levels of NH_4_^+^ concentration also heavily affected the growth of DMSS-1, with ammonium-depleted conditions showing faster csSRR but lower growth yield. This is also consistent with a previous study of DMSS-1 grown on NH_4_^+^ -depleted conditions (18).

### Multiple sulfur isotope fractionation

**Fig 1:**
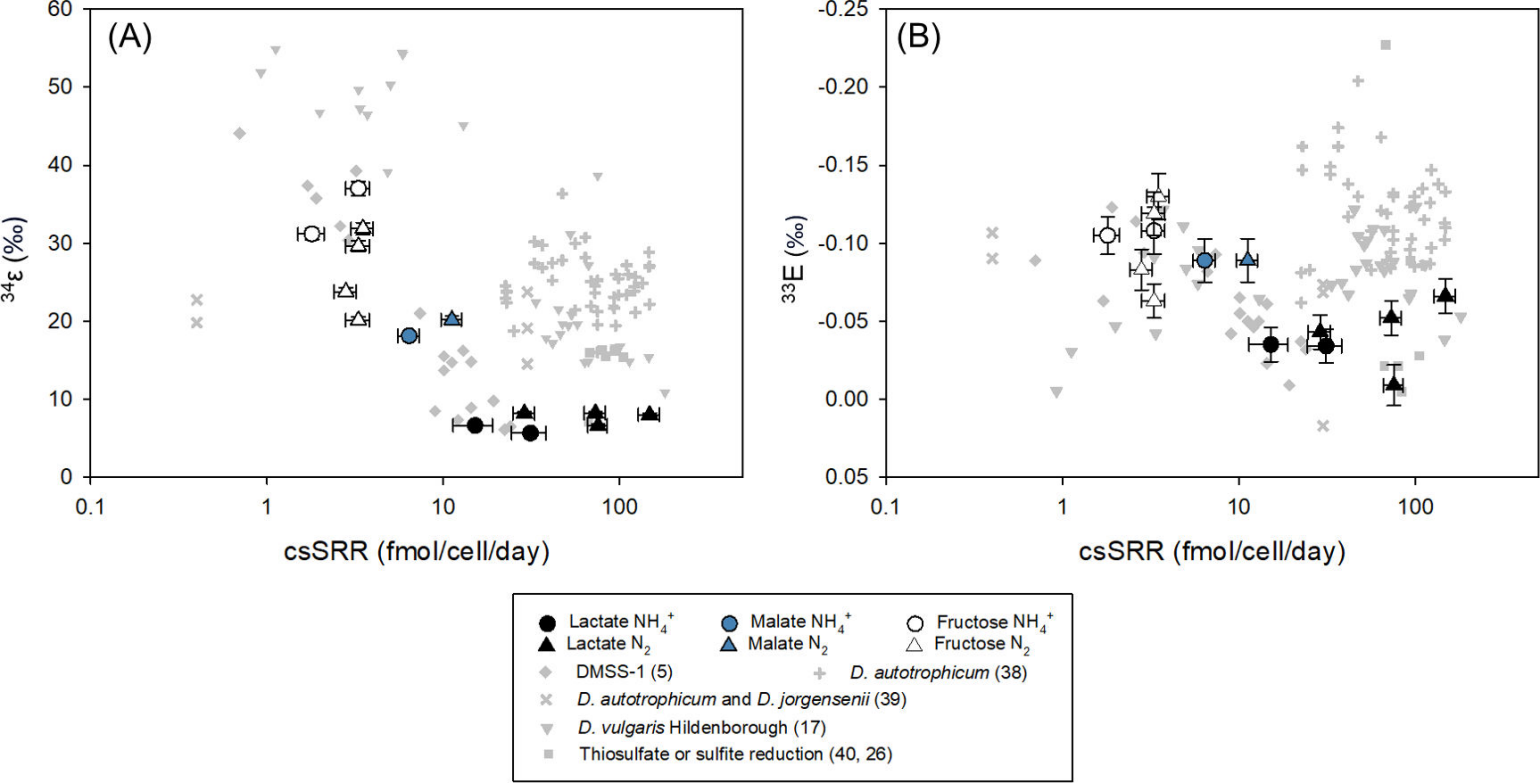
Comparison of multiple sulfur isotope fractionation values and csSRR measured in this study with that of previously published data (5, 17, 26, 38–40). (**A**) ^34^ε vs csSRR. (**B**) ^33^Ε vs csSRR. The overall trend is an inverse correlation between ^34^ε and csSRR, but sulfate reduction with lactate and malate coupled with N_2_ fixation displays an increase in both ^34^ε and csSRR. Some of the ^33^Ε values of previous studies were recalculated according to the equations presented in this study.

DMSS-1 under diverse conditions expressed a wide range of ^34^ε values between 5.7‰ and 37.0‰ (Table 1). The magnitude of ^34^ε for the three different electron donors has been studied under NH_4_^+^-replete conditions before (5), and the results shown here are generally similar to the previously reported values. Lactate produced the smallest ^34^ε, while fructose produced the largest fractionation values, overall displaying an inverse correlation between ^34^ε and csSRR (Fig. 1A). This inverse trend is consistent with findings from previous studies investigating the influence of electron donors (5, 17).

The availability of NH_4_^+^ altered the pattern of ^34^ε as previously reported (18). In both the lactate and malate experiments, ^34^ε slightly increased when DMSS-1 fixed nitrogen for their growth. When utilizing fructose as an electron donor, however, N_2_-fixing DMSS-1 showed increased csSRR but expressed a lower ^34^ε, deviating from the previously reported pattern (18).

Mass-dependent fractionation of ^33^S was observed in the form of ^33^E, ranging from −0.034‰ to −0.105‰. ^33^E and ^34^ε generally showed an inverse correlation (Fig. 1), meaning that the experiment with lactate showed the highest ^33^E values, while that of fructose showed the lowest ^33^E values. Our csSRR–³³E data fall within the range of values reported by previous studies, particularly those examining the effects of electron donors (5, 17), although differences in microbial species and experimental conditions complicate direct comparison across studies.

### Intracellular energy state

**Fig 2:**
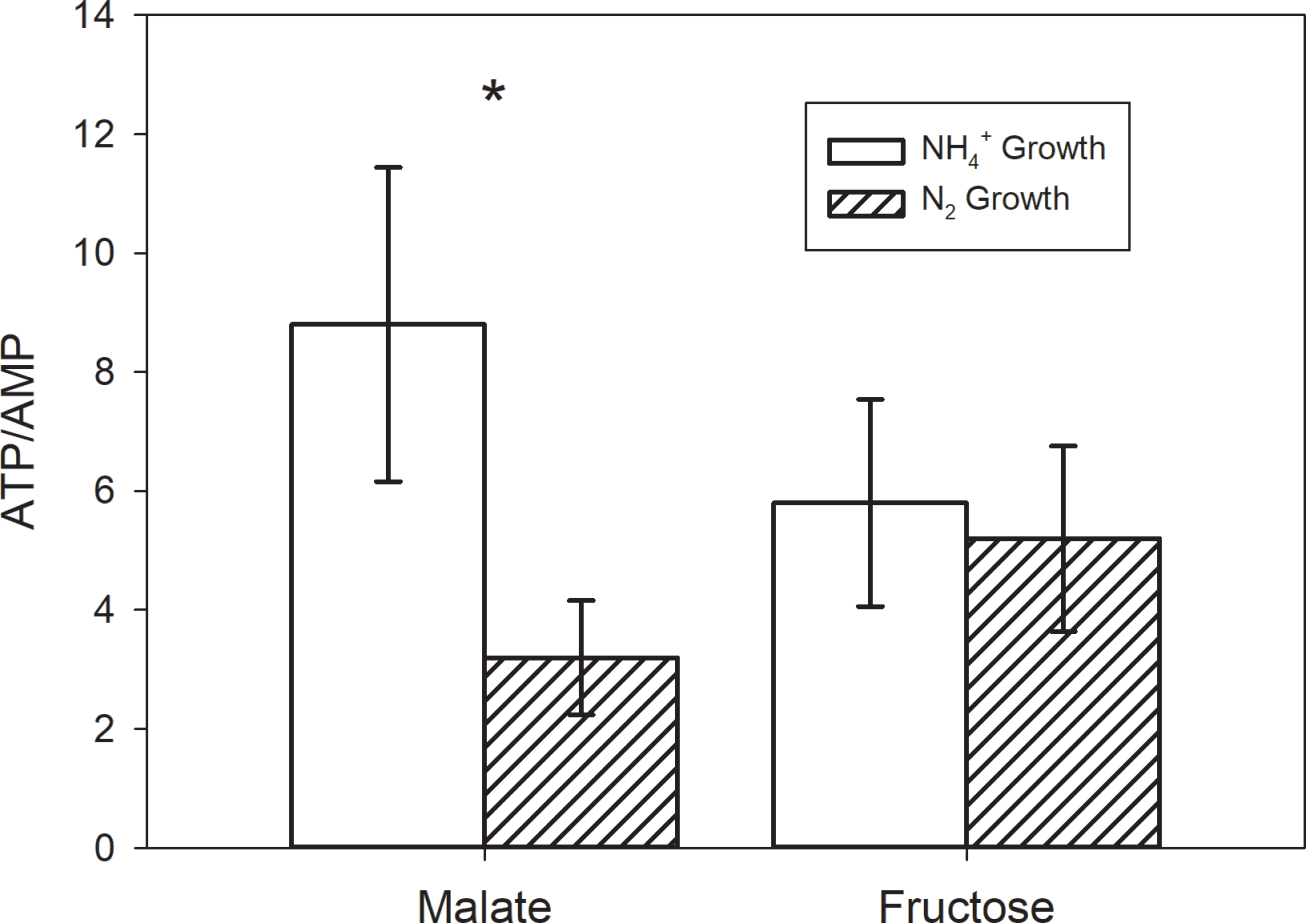
Intracellular ATP/AMP data from malate and fructose batch culture experiments. The uncertainty of the ATP/AMP ratio was derived from the standard deviations of measurements taken for both the prepared standards and batch culture samples (*n* > 5). Asterisk indicates a statistically significant difference between NH_4_^+^ growth and N_2_ growth (*t*-test *P* value < 0.05).

N_2_ fixation is linked to ATP consumption (19). Due to the significant impact ATP has on the favorability (and reversibility) of the sulfate activation step in the sulfate reduction pathway, the energy state (intracellular ATP, ADP, and AMP) of DMSS-1 during malate and fructose consumption was monitored. The experiment with malate showed a significant decrease in ATP/AMP ratio (*t*-test *P* value < 0.05), with NH_4_^+^-replete condition showing a ratio of 8.8, while N_2_-fixing condition dropping the ratio down to 3.2 (Fig. 2). Meanwhile, the ATP/AMP ratio results with fructose as the electron donor displayed a minor difference of 0.6, which was considered statistically insignificant (*t*-test *P* value > 0.05).

### Gene expression level of *aprA* and *dsrA* genes

**Fig 3:**
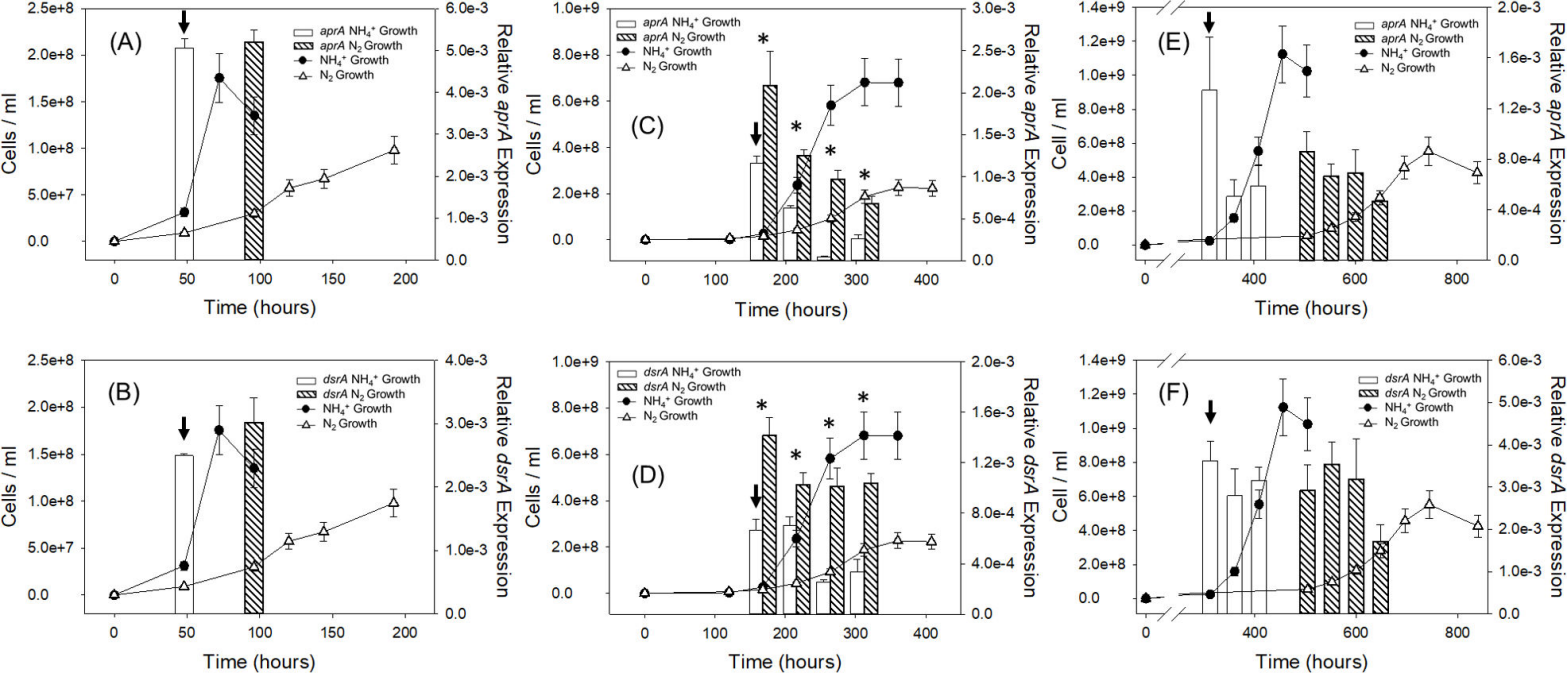
Growth curve of DMSS-1 and relative gene expression levels of *aprA* and *dsrA* under ammonium-replete or deplete conditions. (**A**) Lactate *aprA*. (**B**) Lactate *dsrA*. (**C**) Malate *aprA*. (**D**) Malate *dsrA*. (**E**) Fructose *aprA*. (**F**) Fructose *dsrA*. Shaded bar graphs represent gene expression levels during ammonium-deplete growth, while white bar graphs represent gene expression levels during ammonium-replete growth. Gene expression should be compared only within each panel, where samples were processed and analyzed simultaneously. Black dots represent cell densities during ammonium-replete growth, while white triangles represent cell densities during ammonium-deplete growth. The analytical uncertainty of cell density was ±15%, while the uncertainty of the gene expression analysis was based on the standard deviations of measurements from each of the triplicated batch culture experiments, with each batch culture measured three times (*n* = 9). Black arrows represent samples selected for reanalysis for comparison based on different electron donors under ammonium-replete condition (shown in Fig. 4). Asterisk indicates statistically significant difference between NH_4_^+^ growth and N_2_ growth under similar growth phase (*t*-test *P* value < 0.05; Table S3).

**Fig 4:**
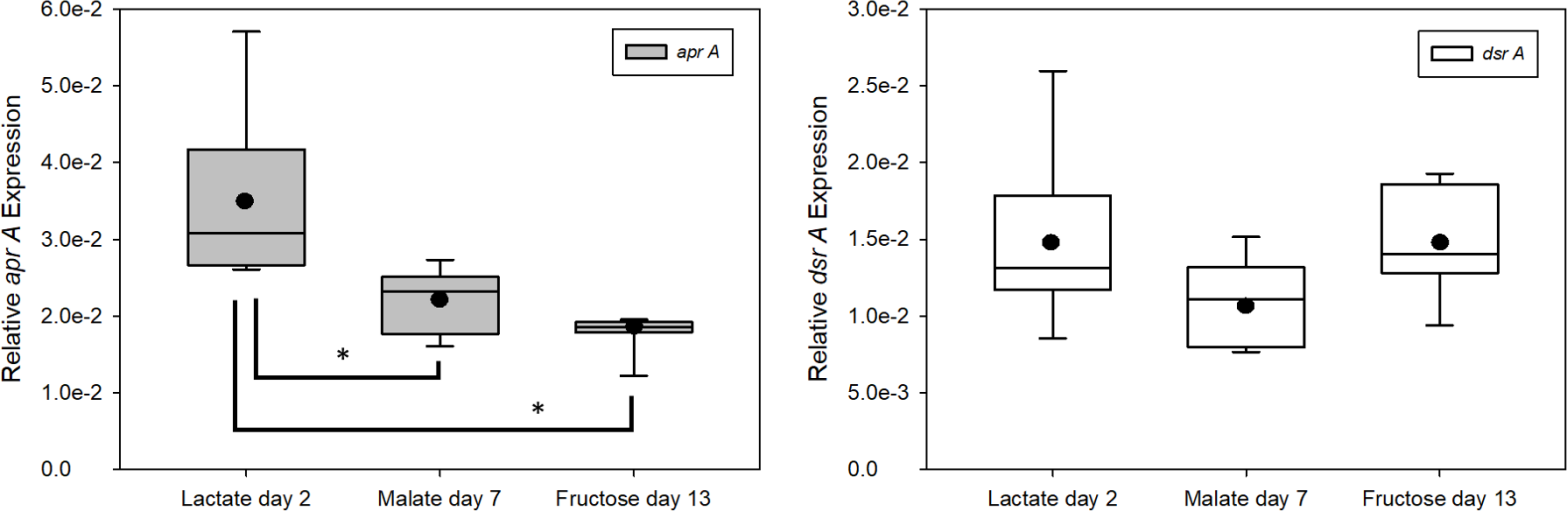
Box-and-whisker plots showing the relative gene expression level of *aprA* and *dsrA* based on different types of electron donors under ammonium-replete condition. Early log-phase samples were selected (marked with arrows in Fig. 3), processed, and analyzed together to ensure fair comparison. Boxes represent the interquartile range with the median shown as a horizontal line, while whiskers represent the range. Black dots indicate the average gene expression values (*n* = 9), and asterisks indicate statistically significant difference between the different electron donors (*t*-test *P* value < 0.05).

Dissimilatory sulfate reduction is a multistep enzymatic process, with adenylyl-sulfate reductase (Apr) and dissimilatory sulfite reductase (Dsr) being the two enzymes that reduce activated sulfate (APS) to sulfite and then to sulfide, respectively. To understand the respiratory activity of DMSS-1 in different growth conditions, RT-qPCR analysis was conducted to see the change in relative gene expression levels of *aprA* and *dsrA,* which encode subunits of these two respective proteins. RT-qPCR was conducted separately for each electron donor experiment, displaying various levels of *aprA* and *dsrA* expression by DMSS-1 at different growth stages and under varying NH_4_^+^ availability (Fig. 3; Table S3). However, the separate experimental sessions made it difficult to compare gene expression levels based on the types of electron donors due to the variability in the reverse transcription (RT) efficiency between each experimental session. Therefore, RNA samples during the early exponential growth phase from each electron donor experiment were selected for reanalysis, aligning the RT-qPCR efficiency for a fair comparison (Fig. 4; Table S4). Changing the electron donor and carbon source of the medium significantly altered the expression level of *aprA* (*t*-test *P* value < 0.05). Lactate consumption showed the highest expression of *aprA*, but the expression level decreased as the electron donors became more difficult to break down, with fructose being the most refractory electron donor (Fig. 4; Table S4). *DsrA,* on the other hand, did not show any statistically significant change throughout the alterations in csSRR or the types of electron donor.

Change in NH_4_^+^ concentration, on the other hand, resulted in an alteration of both *aprA* and *dsrA* gene expression levels. DMSS-1 grown on malate with NH_4_^+^-depleted medium showed a statistically significant increase (*t-*test *P* value < 0.05) in both *aprA* and *dsrA* by at least around 1.8- and 1.5-fold, respectively, with respect to the NH_4_^+^-replete medium (Fig. 3; Table S3). This was rather expected due to the increase of csSRR in growth coupled with N_2_ fixation. However, such a change was not observed in the experiments with fructose.

### Gene expression level of *pyk*, *pfk*, and *gap* genes during fructose consumption

To potentially understand the decreased fractionation factors during the N_2_ fixation experiment with fructose as the electron donor, additional biochemical factors were observed. We measured the expression level of major genes involving the Embden-Meyerhof-Parnas (EMP) pathway to observe the potential change in central metabolism during N_2_ fixation. Three genes were observed: pyruvate kinase (*pyk*), phosphofructokinase (*pfk*), and glyceraldehyde 3-phosphate dehydrogenase (*gap*). *pyk* and *pfk* did not show any statistically significant changes (*t*-test *P* value > 0.05) between N_2_-fixing and NH_4_^+^-replete conditions (Table S2). However, *gap* gene expression showed a significant increase (*t*-test *P* value < 0.05) during N_2_ fixation by about twofold (Fig. S2).

## DISCUSSION

### Effect of electron donors on *aprA* and *dsrA* expression

Gene expression data from experiments using different electron donors showed up to a twofold change in *aprA* expression levels (Fig. 4; Table S4), with higher *aprA* expression associated with faster respiration rates and smaller sulfur isotope effects. The pivotal role of the APS reduction step in shaping dissimilatory sulfur isotope fractionation has been inferred from theoretical modeling (14), oxygen isotope labeling experiments (41), and assessments of the kinetic sulfur isotope effects imparted by APS reductase (30). The reversibility of each enzymatic reaction dictates the magnitude of sulfur isotope fractionation (11, 12). This reversibility, in turn, is thermodynamically constrained by the flux-force relationship, which links it to the actual Gibbs free energy change of the reaction (14, 42). Thus, the observed relationship among *aprA* expression, csSRR, and sulfur isotope fractionation suggests that the expression of APS reductase is likely coupled to the intracellular energy state, corroborating its proposed role in controlling sulfur isotope fractionation. Results from a recent proteomics-based study, which manipulated the availability of organic electron donors rather than their chemical nature, also align well with our observed *aprA* expression patterns (7). Notably, the electron acceptor was not a limiting factor in either study. Given that sulfate reduction is largely controlled by the supply of organic substrates rather than by sulfate availability in most marine habitats (43, 44), the regulation of *aprA* expression in response to changes in organic substrate availability and reactivity may be key to interpreting microbial sulfur isotope fractionation and its imprint in marine porewaters and sedimentary records.

Meanwhile, *dsrA* expression level did not show any statistically significant changes, although previous studies on sulfate reducers have often reported a positive correlation between *dsr* gene expression and csSRR (45, 46). A notable difference here is that those studies demonstrated this correlation by varying the availability or chemistry of the electron acceptors (e.g., thiosulfate), whereas our study maintained a non-limiting supply of sulfate throughout the experiments. Interestingly, large-scale phylogenetic analyses of sulfate reducers suggest that the *dsr* gene cluster was utilized ancestrally before the advent of *apr* (47, 48). This evolutionary sequence is consistent with geological records indicating that bioavailable electron acceptors were scarce on primitive Earth (3, 49), and the availability of such electron acceptors gradually increased through progressive global oxidation (50). Such evolutionary history may be responsible for the distinct regulation of *dsr* and *apr* in response to changes in electron donors and acceptors. In addition, elevated intracellular sulfite levels are known to disrupt cellular functions by interfering with redox homeostasis (51) and inhibit bacterial growth (52, 53). While the continuous expression of *dsr* is most likely driven by energy conservation, it may also serve to mitigate the inhibitory effects of sulfite accumulation. However, further studies on microbial physiology and proteome allocation will be required to elucidate the mechanisms underlying the stable expression of *dsrA*.

Comparatively, *apr* appears to play a greater role than *dsr* not only in determining the magnitude of sulfur isotope fractionation but also in driving its inverse correlation with csSRR. Based on the results presented here and those of previous studies, the type of electron donor (this study) or its availability (7) can alter the expression of *apr* and bring about the well-established inverse correlation between csSRR and ^34^ε. Meanwhile, the availability of sulfate affects the expression of *dsr* (45, 46), which does not lead to the inverse csSRR-^34^ε relationship (6, 54–56). Hence, the broad inverse trend observed in pure culture studies over the past seven decades, where maximum sulfur isotope fractionation decreases sharply with increasing csSRR (56), suggests that changes in sulfur dissimilation rates and isotope effects are largely governed by Apr, which operates upstream of Dsr in the dissimilatory sulfate reduction pathway.

### The effect of N_2_ fixation on microbial sulfur isotope fractionation

Consistent with a previous report (18), diazotrophic growth increased both csSRR and ^34^ε when lactate and malate were used as electron donors. The observable changes in gene expression and ATP/AMP ratios in this study experimentally support the speculation by Sim et al. (18): the simultaneous increase in csSRR and ^34^ε during diazotrophic growth may result from higher metabolic fluxes coupled with increased reversibility of individual enzymatic steps. In experiments with malate as an electron donor, elevated expression of both *aprA* and *dsrA* may have increased metabolic fluxes, and the highly energy-demanding N₂ fixation process led to a decrease in the ATP/AMP ratio, making the sulfate activation and APS reduction steps more reversible.

**Fig 5:**
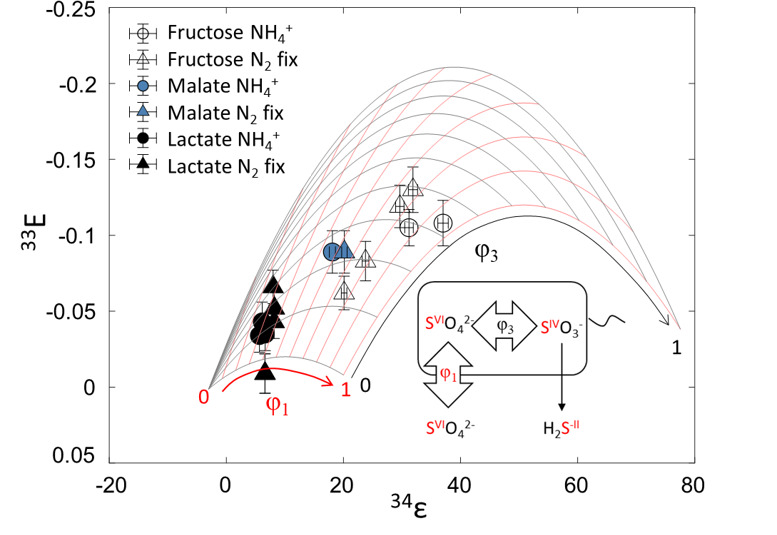
Metabolic flux model assuming that sulfate reduction is a two-step reversible process. Isotope fractionation data obtained from batch culture experiments are plotted inside the model, indicating that the results are all (except one) explainable through sulfate reduction. Diagram shown on the bottom right corner depicts the metabolic steps considered in this model. φ_1_ represents the reversibility of the first step, which is the transport of sulfate into the cell. φ_3_ represents the reversibility of the reduction of intracellular sulfate into sulfite. Note that under diazotrophic conditions, φ_1_ decreased in the fructose experiments, whereas the opposite trend was observed with malate and lactate. Error bars shown here are based on the propagations of analytical uncertainties of sulfide, sulfate, and isotope measurements.

In contrast, our new findings with fructose as an electron donor appear to be inconsistent with this earlier hypothesis. Although csSRR increased slightly in response to N_2_ fixation, neither RT-qPCR nor ATP/AMP assay results showed significant differences between N₂-fixing and NH₄^+^-grown cells (Fig. 2 and 3). The invariant ATP/AMP ratios may be ascribed to the fact that fructose, a more energy-rich substrate (see ${∆G}_{{}^{\circ}}^{`}$ values shown in Materials and Methods), yields more ATP through glycolysis via substrate-level phosphorylation compared to malate. Previous studies have shown that N₂ fixation does not always alter the relative ratios of adenosine phosphates, depending on the balance between ATP regeneration and cellular energy demand (57). Thus, with fructose as the organic substrate, N_2_-fixing cells might accelerate fructose catabolism to generate additional ATP without upregulating sulfur reduction genes. The ability of DMSS-1 to ferment carbohydrates may enable this metabolic decoupling between carbon and sulfur catabolism (58). However, this explanation alone is insufficient to account for the decreased ³⁴ε values observed during diazotrophic growth. In particular, applying a simplified metabolic flux model that treats sulfate reduction as a two-step reversible process (11, 13) to our multiple sulfur isotope data reveals a distinct pattern (Fig. 5): under diazotrophic conditions, leakage of sulfate across the cell membrane decreased in the fructose experiments, whereas the opposite trend was observed with malate and lactate. That is, the impact of N_2_ fixation on the overall reversibility of the microbial sulfate reduction pathway is distinct in fructose-grown cultures.

One hypothesis for the less reversible sulfate reduction, despite unchanged expression of sulfur reduction genes and intracellular energy state, is that accelerated fructose catabolism driven by increased ATP demand shifts the intracellular redox potential to a more negative value, thereby limiting the reversibility of the overall sulfate reduction pathway. While the specific metabolic pathway of DMSS-1 has not yet been elucidated, fructose-utilizing, sulfate-reducing bacteria have been previously suggested to use the EMP pathway (59, 60). Thus, we monitored the expression of three genes involved in glycolysis: *pyk*, *pfk*, and *gap* (Table S2). *Pyk* and *pfk* did not show a statistically significant difference between the two different nitrogen growth conditions, but *gap* gene expression increased by about twofold (*t*-test *P* value < 0.05) under the diazotrophic condition (Fig. S2). Although it is difficult to conclude that the overall glycolytic rate increased in N₂-fixing DMSS-1 cells, the significant upregulation of *gap* may be associated with a shift in the intracellular redox state. The enzyme glyceraldehyde 3-phosphate dehydrogenase (GAPDH), encoded by *gap*, not only participates as a part of the EMP pathway but also functions in the reduction of nicotinamide adenine dinucleotide (NADH). While GAPDH is often used experimentally as a housekeeping gene (61), its expression level can vary under certain conditions (62, 63). Interestingly, elevated *gap* gene expression or increased GAPDH abundance has been associated with higher NADH/NAD^+^ ratios in some bacterial strains (64–66). If this is the case for DMSS-1, an increase in NADH concentration could enhance electron availability, potentially leading to less reversible sulfate respiration in diazotrophic cells using fructose as the electron donor.

One understudied aspect of microbial sulfate reduction is the intracellular redox state, which warrants further investigation. Given that N_2_ fixation requires both ATP and electron donors, it is logical to expect that it would act as a sink for reducing power. However, our isotopic results with fructose do not appear to support this. Observing other isotopes, such as the deuterium/protium (^2^H/^1^H) ratio of microbial lipids, may provide additional information on the intracellular production of NADPH (67, 68). Direct measurement of intracellular redox state using electron carriers, such as the NAD^+^/NADH couple, could provide clearer evidence, although the dominant carriers may shift depending on environmental conditions (14, 69).

### Relationship between csSRR and ^34^ε in natural habitats

**Fig 6:**
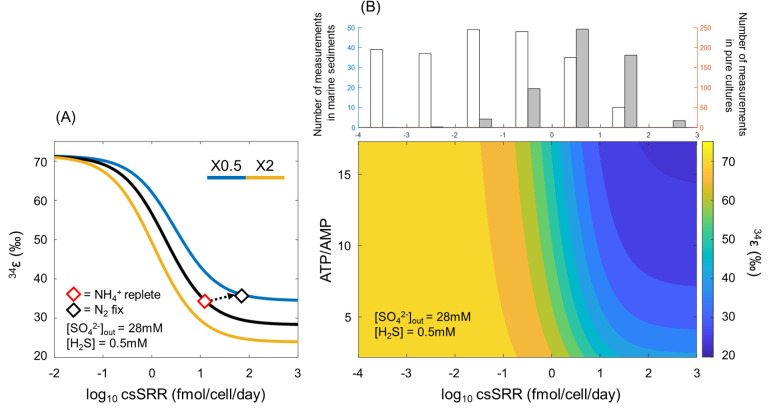
Microbial sulfur isotope model predicting the sulfur isotope fractionation pattern depending on the energy state of the cell. The original model by Wing and Halevy (14) was modified using altering intracellular adenosine profiles. (**A**) Black line represents the original model with default parameters proposed by Wing and Halevy (14). The yellow and blue line simulates half and twice the default ATP/AMP ratio value, respectively. In a relatively high range of csSRR, both ^34^ε and csSRR can increase simultaneously as the ATP/AMP ratio decreases (red vs black diamond). (**B**) Contour map showing more detailed changes in sulfur isotope fractionation under different ATP/AMP ratios and csSRRs. The range of log-scale csSRR is based on the previously reported values in natural and laboratory settings (16). For the range of ATP/AMP ratio, the minimum value was chosen based on the lowest values measured for anaerobic bacteria to date (70–72), while the maximum value was selected as twice the default value used in Wing and Halevy (14). Histograms of csSRR values measured in marine sediments (white) and laboratory pure cultures (gray), compiled from Jørgensen et al. (16) and Sim et al. (56). Note that the majority of csSRR values measured in marine sediments are below 1 fmol/cell/day.

In this study, the relationship between csSRR and ^34^ε varied dynamically, depending on the availability of fixed nitrogen and the type of organic substrate. A simultaneous increase in both csSRR and ^34^ε was observed during malate consumption coupled with N_2_ fixation, representing a notable exception to the typical inverse relationship. On the other hand, diazotrophic growth with fructose as an organic substrate led to increased csSRR but decreased ^34^ε, consistent with the conventional negative correlation. Intracellular ATP/AMP ratios also varied depending on physiological conditions. To account for these observations, we applied the metabolic flux model for microbial sulfur isotope fractionation (14). The model, assuming steady state of sulfur-bearing reactants and products inside the cell, determines the net isotope fractionation using fractionation factors and reversibility of each sulfate reduction step. The reversibility of each step can be defined using the flux-force relationship, mathematically linking it with the actual free energy change of the step, which is calculated with various experimentally accessible biochemical parameters such as enzyme kinetics and electron carrier concentration (14). Although the original model incorporates a broad range of biological parameters, it assumes several, including intracellular ATP and AMP concentrations, as invariant, except during sensitivity analyses. Accordingly, we modified the model using new experimental constraints on intracellular ATP/AMP ratios (Fig. 6A). The overall relationship between csSRR and ³⁴ε remained intact across different intracellular energy states, but the curve shifted vertically depending on the ATP/AMP ratio. When N_2_ fixation was simulated (i.e., a 0.5× decrease in the ATP/AMP ratio), the model predicted a general increase in ³⁴ε across nearly the entire range of csSRR, reproducing a scenario similar to our culture experiment with malate as the electron donor, where diazotrophic growth resulted in higher ³⁴ε despite a higher csSRR compared to ammoniotrophic growth. The effect of varying ATP/AMP ratios on model outcomes became increasingly pronounced at higher csSRR values, where the slope of the model curve progressively declined.

Our results suggest that microbial sulfur isotope fractionation can be uniquely influenced by combinations of environmental factors, including carbon source and nitrogen availability, making the interpretation of natural sulfur isotope records a complex task. It is well established from various previous studies, however, that csSRR in natural environments is substantially lower than in laboratory settings primarily due to the scarcity of available organic substrates (16). This corresponds to the model results when csSRR approaches below 1 fmol/cell/day in Fig. 6A, where the slopes of the model curves steepen dramatically, and the impact of varying ATP/AMP ratios becomes diminished. A more detailed pattern is provided in the contour plot (Fig. 6B), where ^34^ε changes as a function of both csSRR and ATP/AMP ratio. At relatively high csSRR values (1–10^2^ fmol/cell/day), ^34^ε can be modulated by the adenosine phosphate levels, potentially explaining cases where both ³⁴ε and csSRR increase during N_2_ fixation. However, when csSRR drops below 1 fmol/cell/day, ³⁴ε becomes largely insensitive to changes in ATP/AMP ratio, and csSRR emerges as the dominant control. A majority of the csSRR values reported in natural environments, such as marine sediments, are well below this threshold, whereas those of culture experiments typically range from 1 to 10^2^ fmol/cell/day (Fig. 6B). As a result, this study goes beyond exploring the mechanisms behind the variable relationship between csSRR and ^34^ε under different physiological conditions. The clear contrast observed between the malate- and fructose-grown cultures, combined with insights from the metabolic flux model, suggests that the well-established inverse correlation between csSRR and ³⁴ε is likely to remain robust in natural environments where sulfate reduction proceeds slowly.

### Conclusion

The sulfate-reducing bacterium DMSS-1 was cultivated with different electron donors and under varying fixed nitrogen conditions in order to explore dynamic changes in the relationship between csSRR and sulfur isotope fractionation. While the apparent influence of N_2_ fixation has been described previously, this study provides a biochemical basis that helps explain the mechanisms underlying these changes. When utilizing simple organic acids, N_2_-fixing cells showed increases in both csSRR and ^34^ε. Changes in gene expression and adenosine phosphate levels suggest that elevated expression of *aprA* enhances metabolic sulfur fluxes, while a lower ATP/AMP ratio may render sulfate activation and APS reduction more reversible, together contributing to a net increase in both csSRR and ^34^ε. In contrast, gene expression and adenosine phosphate levels remained largely unaltered in fructose-grown cultures regardless of nitrogen source, although ³⁴ε decreased significantly under diazotrophic conditions, accompanied by a slight increase in csSRR. A multiple sulfur isotope model suggests that less sulfate leaked from the cell, and upregulation of the *gap* gene further supports the interpretation of a less reversible sulfate reduction pathway under N_2_ fixation, with the Gap enzyme potentially delivering NADH into the redox pool as fructose is oxidized. Overall, microbial sulfur isotope effects should not be viewed merely as a simple inverse function of csSRR, but rather as an emergent outcome of multiple, interconnected intracellular processes. However, in most natural environments, labile organic carbon is rapidly consumed aerobically, leaving only more recalcitrant substrates for sulfate reducers (16). As such, the reversal of the typical inverse csSRR-^34^ε relationship observed in experiments with simple organic acids may be extremely rare in natural settings, as also suggested by our modified sulfur isotope model for MSR.

## Data Availability

All data used to reach the conclusions in the paper are present in the paper and/or the supplemental material.

## SUPPLEMENTAL MATERIAL

Supplemental material aem.01064-25-s0001.docx Tables S1 to S4; Fig. S1 and S2.

ASM does not own the copyrights to Supplemental Material that may be linked to, or accessed through, an article. The authors have granted ASM a non-exclusive, world-wide license to publish the Supplemental Material files. Please contact the corresponding author directly for reuse.
