# Supplementary material for: Molecular insights into the dynamic relationship between respiration rate and sulfur isotope effect
Source: Appl Environ Microbiol. 2025 Oct 16;91(11):e01064-25. doi: 10.1128/aem.01064-25 (PMC12628773; doi:10.1128/aem.01064-25)
Supplement: Supplemental material — Tables S1 to S4; Fig. S1 and S2. [file aem.01064-25-s0001.docx]

**Supplementary Material for**

**Molecular insights into the dynamic relationship between respiration rate and sulfur isotope effect**

Dong Kyun Woo ^a*^, Bokyung Kim ^a*^, Yuichiro Ueno ^b,c^, Shawn E. McGlynn ^c,d,e^, Min Sub Sim ^a#^

*^a^ School of Earth and Environmental Sciences, Seoul National University, Seoul 08826, South Korea*

*^b^ Department of Earth and Planetary Sciences, Institute of Science Tokyo, Meguro, Tokyo 152-8551, Japan*

*^c^ Earth-Life Science Institute, Institute of Science Tokyo, 2-12-1-IE-1 Ookayama, Meguro-ku, Tokyo 152-8550, Japan*

*^d^ Blue Marble Space Institute of Science, Seattle, WA, USA*

*^e^ Biofunctional Catalyst Research Team, RIKEN Center for Sustainable Resource Science, Wako, Japan*

^#^For correspondence

**Min Sub Sim**

School of Earth and Environmental Sciences

Seoul National University

Room 606, Building 25-1

1 Gwanak-ro, Gwanak-gu, Seoul 08826, South Korea

Tel) +82 2 880 6632

Email) [mssim@snu.ac.kr](mailto:mssim@snu.ac.kr)

*Dong Kyun Woo and Bokyung Kim contributed equally to this manuscript.

| **Table S1.** List of primers and their PCR method used in this study   \| Target gene \| Primer name \| Primer sequence (5'-3') \| PCR method \| Average \| Primer \| Amplicon  length \| Reference \| \| --- \| --- \| --- \| --- \| --- \| --- \| --- \| --- \| \| efficiency \| concentration \| \| *apr A* \| Aps-3f \| TGGCAGATCATGWTYAAYGG \| Hot start step^1^: 50°C for 2 min → 95°C for 2min \| 87.40% \| 50µM \| 367 bp \| (1) \| \|  \| Aps-2 \| GGGCCGTAACCRTCYTTRAA \| PCR step: 40 cycles of 95°C for 15 sec → 58°C for 1 min → 72°C for 1 min \| \|  \|  \|  \|  \|  \|  \|  \|  \| \| *dsr A* \| Dsr-1F \| ACSCACTGGAAGCACGCCGG \| PCR step: 40 cycles of 95°C for 15 sec → 60°C for 1 min \| 84.70% \| 20µM \| 184 bp \| (2) \| \|  \| Dsr-R \| GTGGMRCCGTGCAKRTTGG \| \|  \|  \|  \|  \|  \|  \|  \|  \| \| *16s* \| Eub341F \| CCTACGGGAGGCAGCAG \| PCR step: 40 cycles of 95°C for 15 sec → 60°C for 1 min \| 87.60% \| 20µM \| 192 bp \| (3) \| \| *rRNA* \| Eub534R \| ATTACCGCGGCTGCTGGC \| \|  \|  \|  \|  \|  \|  \|  \|  \| \| *pyk* \| pyk-f \| ATTGTGGCTACCCAGATGCT \| PCR step: 40 cycles of 95°C for 15 sec → 55°C for 1 min → 72°C for 1 min \| 89.25% \| 20µM \| 125 bp \| (4, 5)^2^ \| \|  \| pyk-r \| GGCAGTTTCTTCGGAAAGC \| \|  \|  \|  \|  \|  \|  \|  \|  \| \| *pfk* \| pfk-f \| ATCAGCATCCTGTTCGTCAT \| PCR step: 40 cycles of 95°C for 15 sec → 55°C for 1 min → 72°C for 1 min \| 90.05% \| 20µM \| 126 bp \| (4, 5)^2^ \| \|  \| pfk-r \| TGTCGTTGTCAATGGTCTTG \| \|  \|  \|  \|  \|  \|  \|  \|  \| \| *gap* \| gap-f \| CACGATCCACTCCTACACCA \| PCR step: 40 cycles of 95°C for 15 sec → 55°C for 1 min → 72°C for 1 min \| 90.07% \| 20µM \| 331 bp \| (4, 5)^2^ \| \|  \| gap-r \| CGATAAAGTCGGACGAAACC \| |
| --- | --- | --- | --- | --- | --- | --- | --- | --- | --- | --- | --- | --- | --- | --- | --- | --- | --- | --- | --- | --- | --- | --- | --- | --- | --- | --- | --- | --- | --- | --- | --- | --- | --- | --- | --- | --- | --- | --- | --- | --- | --- | --- | --- | --- | --- | --- | --- | --- | --- | --- | --- | --- | --- | --- | --- | --- | --- | --- | --- | --- | --- | --- | --- | --- | --- | --- | --- | --- | --- | --- | --- | --- | --- | --- | --- | --- | --- | --- | --- | --- | --- | --- | --- | --- | --- | --- | --- | --- | --- | --- | --- | --- | --- | --- | --- | --- | --- | --- | --- | --- | --- | --- | --- | --- | --- | --- | --- | --- | --- | --- | --- | --- | --- | --- | --- | --- | --- |

^1^Hot start step is based on the manual from Power Up Sybr green qPCR master mix. The same step is applied for the rest of the other target genes as well.
^2^Primer was based on DMSS-1 genome sequence with annotation available on The Genome Portal of the Department of Energy Joint Genome Institute Project ID: 1012234 (4). Primer design was done by using PrimerIdent (5).

**Table S2.** Relative gene expression levels of glycolysis genes during fructose consumption by DMSS-1. NH_4_^+^ and N_2_ fix data were compared based on similar growth stages including the lag, early exponential, and mid exponential phase. FC represents fold change (N_2_ fix / NH_4_^+^). *P* values lower than 0.05 were considered statistically significant.

| Target gene | Early exponential phase | |  |  | Early exponential phase 2 | |  |  | Mid exponential phase | |  |  |
| --- | --- | --- | --- | --- | --- | --- | --- | --- | --- | --- | --- | --- |
|  | Day 13  (NH_4_^+^) | Day 21  (N_2_ fix) | FC | T test  *p* value | Day 15  (NH_4_^+^) | Day 23  (N_2_ fix) | FC | T test  *p* value | Day 17  (NH_4_^+^) | Day 25  (N_2_ fix) | FC | T test  *p* value |
| *pfk* | 2.81×10^-4^ | 3.05×10^-4^ | 1.086 | 0.772 | 2.25×10^-4^ | 2.98×10^-4^ | 1.322 | 0.208 | 3.39×10^-4^ | 5.93×10^-4^ | 1.750 | 0.098 |
|  |  |  |  |  |  |  |  |  |  |  |  |  |
| *pyk* | 1.51×10^-3^ | 1.16×10^-3^ | 0.769 | 0.317 | 8.82×10^-4^ | 8.39×10^-4^ | 0.951 | 0.896 | 1.18×10^-3^ | 1.38×10^-3^ | 1.165 | 0.437 |
|  |  |  |  |  |  |  |  |  |  |  |  |  |
| *gap* | 7.38×10^-4^ | 1.05×10^-3^ | 1.417 | 0.328 | 5.85×10^-4^ | 1.19×10^-3^ | 2.036 | 0.083 | 9.64×10^-4^ | 1.93×10^-3^ | 2.006 | 0.027 |

**Table S3.** Comparison of average relative gene expression based on different NH_4_^+^ conditions: results from similar growth phase are compared (data from Figure 3)

| **Lactate** | Early exponential phase | | | | | |  |  |  |  |  |  |
| --- | --- | --- | --- | --- | --- | --- | --- | --- | --- | --- | --- | --- |
| Gene | Gene expression | | | | Fold change | |  |  |  |  |  |  |
|  | Day 2 (NH_4_^+^) | Std dev. | Day 4 (N_2_ fix) | Std dev. | (N_2_ fix/  NH_4_^+^) | T test  p value |  |  |  |  |  |  |
| *apr A* | 5.06×10^-3^ | 2.36×10^-4^ | 5.20×10^-3^ | 2.83×10^-4^ | 1.029 | 0.602 |  |  |  |  |  |  |
| *dsr A* | 2.50×10^-3^ | 3.46×10^-5^ | 3.02×10^-3^ | 3.90×10^-4^ | 1.210 | 0.149 |  |  |  |  |  |  |
|  |  |  |  |  |  |  |  |  |  |  |  |  |
| **Malate** | Early exponential phase | | | | | | Mid exponential phase | | | | | |
| Gene | Gene expression | | | | Fold change | | Gene expression | | | | Fold change | |
|  | Day 7 (NH_4_^+^) | Std dev. | Day 7 (N_2_ fix) | Std dev. | (N_2_ fix/  NH_4_^+^) | T test  p value | Day 9 (NH_4_^+^) | Std dev. | Day 9 (N_2_ fix) | Std dev. | (N_2_ fix/  NH_4_^+^) | T test  p value |
| *apr A* | 1.16×10^-3^ | 9.07×10^-5^ | 2.09×10^-3^ | 4.05×10^-4^ | 1.806 | 0.034 | 6.31×10^-4^ | 2.42×10^-5^ | 1.25×10^-3^ | 6.46×10^-5^ | 1.985 | 2.19×10^-4^ |
| *dsr A* | 6.69×10^-4^ | 8.41×10^-5^ | 1.42×10^-3^ | 1.42×10^-4^ | 2.118 | 3.08×10^-3^ | 7.04×10^-4^ | 6.75×10^-5^ | 1.03×10^-3^ | 9.28×10^-5^ | 1.456 | 0.017 |
|  |  |  |  |  |  |  |  |  |  |  |  |  |
| **Malate** | Late exponential phase | | | | | | Stationary phase | | | | | |
| Gene | Gene expression | | | | Fold change | | Gene expression | | | | Fold change | |
|  | Day 11 (NH_4_^+^) | Std dev. | Day 11 (N_2_ fix) | Std dev. | (N_2_ fix/  NH_4_^+^) | T test  p value | Day 13 (NH_4_^+^) | Std dev. | Day 13 (N_2_ fix) | Std dev. | (N_2_ fix/  NH_4_^+^) | T test  p value |
| *apr A* | 4.53×10^-5^ | 6.58×10^-6^ | 9.70×10^-4^ | 1.05×10^-4^ | 21.383 | 2.41×10^-4^ | 2.58×10^-4^ | 5.39×10^-5^ | 6.83×10^-4^ | 8.28×10^-5^ | 2.641 | 3.72×10^-3^ |
| *dsr A* | 2.56×10^-4^ | 1.44×10^-5^ | 1.01×10^-3^ | 1.51×10^-4^ | 3.946 | 2.14×10^-3^ | 3.36×10^-4^ | 9.83×10^-5^ | 1.04×10^-3^ | 7.54×10^-5^ | 3.088 | 1.32×10^-3^ |

**Table S3.** Comparison of average relative gene expression based on different NH_4_^+^ conditions: results from similar growth phase are compared (data from Figure 3 cont.)

| **Fructose** | Early exponential phase | | | | | | Early exponential phase 2 | | | | | |
| --- | --- | --- | --- | --- | --- | --- | --- | --- | --- | --- | --- | --- |
| Gene | Gene expression | | | | Fold change | | Gene expression | | | | Fold change | |
|  | Day 13 (NH4+) | Std dev. | Day 21 (N2 fix) | Std dev. | (N2 fix/  NH4+) | T test  p value | Day 15 (NH4+) | Std dev. | Day 23 (N2 fix) | Std dev. | (N2 fix/  NH4+) | T test  p value |
| *apr A* | 1.35×10^-3^ | 4.19×10^-4^ | 8.56×10^-4^ | 1.60×10^-4^ | 0.636 | 0.198 | 5.05×10^-4^ | 1.33×10^-4^ | 6.66×10^-4^ | 9.44×10^-5^ | 1.319 | 0.235 |
| *dsr A* | 3.63×10^-3^ | 4.67×10^-4^ | 2.92×10^-3^ | 6.12×10^-4^ | 0.805 | 0.263 | 2.80×10^-3^ | 6.43×10^-4^ | 3.54×10^-3^ | 5.15×10^-4^ | 1.268 | 0.268 |
|  |  |  |  |  |  |  |  |  |  |  |  |  |
| **Fructose** | Mid exponential phase | | | | | | Late exponential phase | | | | | |
| Gene | Gene expression | | | | Fold change | | Gene expression | | | | Fold change | |
|  | Day 17 (NH4+) | Std dev. | Day 25 (N2 fix) | Std dev. | (N2 fix/  NH4+) | T test  p value | Day 19 (NH4+) | Std dev. | Day 27 (N2 fix) | Std dev. | (N2 fix/  NH4+) | T test  p value |
| *apr A* | 5.91×10^-4^ | 1.62×10^-4^ | 6.92×10^-4^ | 1.81×10^-4^ | 1.172 | 0.586 | - | - | 4.69×10^-4^ | 5.62×10^-5^ | - | - |
| *dsr A* | 3.15×10^-3^ | 3.16×10^-4^ | 3.18×10^-3^ | 9.60×10^-4^ | 1.011 | 0.965 | - | - | 1.72×10^-3^ | 3.94×10^-4^ | - | - |

**Table S4.** Comparison of average relative gene expression based on different electron donor: results from early exponential phase are reanalyzed for fair comparison (data from Figure 4)

| Gene | Gene expression | | | | | | Fold change | | | |
| --- | --- | --- | --- | --- | --- | --- | --- | --- | --- | --- |
|  | Lactate  day 2 (NH4+) | Std dev. | Malate  day 7 (NH4+) | Std dev. | Fructose  day 13 (NH4+) | Std dev. | (Lactate/  Malate) | T test  p value | (Lactate/ Fructose) | T test  p value |
| *apr A* | 3.48×10^-2^ | 1.01×10^-2^ | 2.21×10^-2^ | 3.90×10^-3^ | 1.78×10^-2^ | 2.35×10^-3^ | 1.574 | 4.87×10^-3^ | 1.952 | 1.38×10^-3^ |
| *dsr A* | 1.49×10^-2^ | 4.84×10^-3^ | 1.08×10^-2^ | 2.62×10^-3^ | 1.47×10^-2^ | 3.20×10^-3^ | 1.375 | 0.054 | 1.009 | 0.955 |


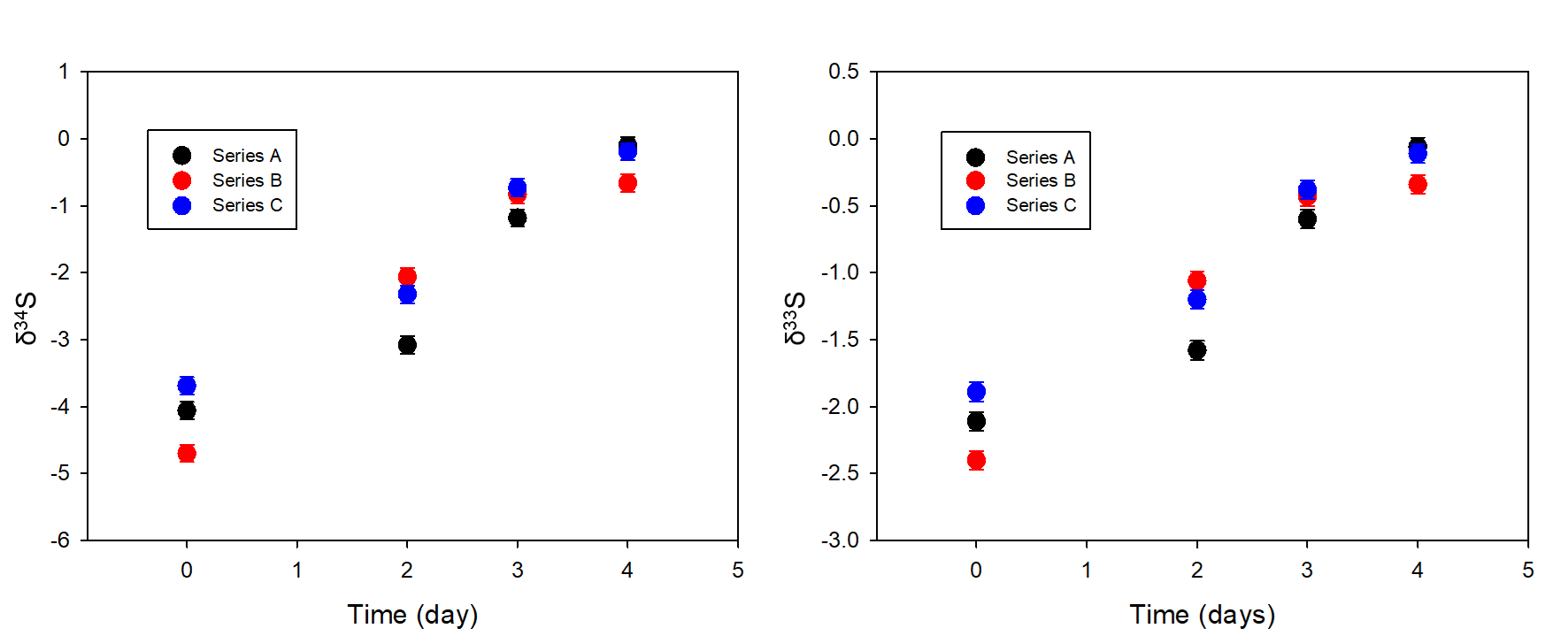
**Figure S1.** Triplicated sulfate isotope data from the NH_4_^+^ replete lactate batch culture experiment. The data shown here were used to confirm the consistency of the isotope measurements displayed in Table 1.

**Figure S2.** Relative gene expression of *gap* gene during fructose consumption by DMSS-1. Asterisk represents statistically significant (P value < 0.05) difference between NH_4_^+^ and N_2_ fix growth. NH_4_^+^ and N_2_ fix data were compared based on similar growth stages including the lag, early exponential, and mid exponential growth phase.


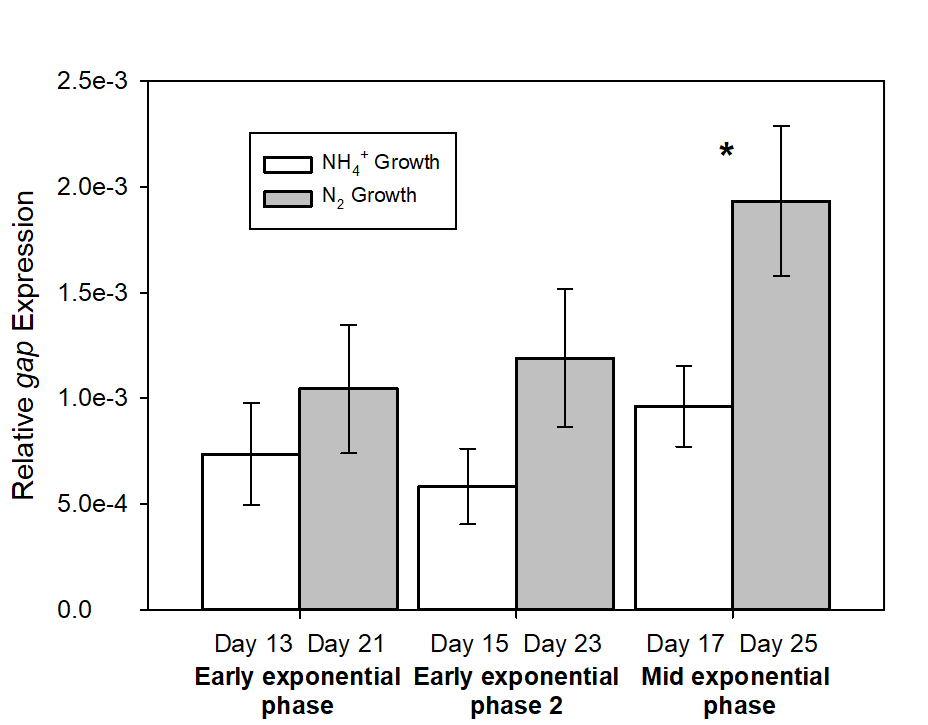


**References**

1. Christophersen, C. T., Morrison, M., Conlon, M. A. 2011. Overestimation of the abundance of sulfate-reducing bacteria in human feces by quantitative PCR targeting the Desulfovibrio 16S rRNA gene. Applied and environmental microbiology, 77(10), 3544-3546.

2. Kondo, R., Shigematsu, K., Butani, J. 2008. Rapid enumeration of sulphate-reducing bacteria from aquatic environments using real-time PCR. Plankton and Benthos Research, 3(3), 180-183.

3. Smits, T. H., Devenoges, C., Szynalski, K., Maillard, J., Holliger, C. 2004. Development of a real-time PCR method for quantification of the three genera Dehalobacter, Dehalococcoides, and Desulfitobacterium in microbial communities. Journal of Microbiological Methods, 57(3), 369-378.

4. Nordberg, H., Cantor, M., Dusheyko, S., Hua, S., Poliakov, A., Shabalov, I., Smirnova, T., Grigoriev, I. V., Dubchak, I. 2014. The genome portal of the Department of Energy Joint Genome Institute: 2014 updates. Nucleic acids research, 42(D1), D26-D31.

5. Pessoa, A. M., Pereira, S., Teixeira, J. 2010. PrimerIdent: a web based tool for conserved primer design. Bioinformation, 5(2), 52.
